# Supplementary material for: Effects of Psychostimulants and Antipsychotics on Serum Lipids in an Animal Model for Schizophrenia
Source: Biomedicines. 2021 Feb 26;9(3):235. doi: 10.3390/biomedicines9030235 (PMC7996855; doi:10.3390/biomedicines9030235)
Supplement: Supplementary file 1 [file biomedicines-09-00235-s001.pdf]

Supplementary information

Effects of psychostimulants and antipsychotics on serum lipids in an animal model for schizophrenia

Banny Silva Barbosa Correia[1], João Victor Nani[2,3] Raniery Waladares Ricardo[1], Danijela Stanisic[1], Tássia Brena Barroso Carneiro Costa[1], Mirian A. F. Hayashi[2,3]* and Ljubica Tasic[1]*

[1] Instituto de Química, Universidade Estadual de Campinas, Campinas, SP, Brazil; ljubica@unicamp.br*

[2] Departamento de Farmacologia, Escola Paulista de Medicina (EPM), Universidade Federal de São Paulo (UNIFESP), São Paulo, SP, Brazil; mhayashi@unifesp.br or mirianhayashi@yahoo.com*

[3] National Institute for Translational Medicine (INCT-TM, CNPq), São Paulo, SP, Brazil; mhayashi@unifesp.br or mirianhayashi@yahoo.com*

Correspondence:
ljubica@unicamp.br; Tel: +55-19-3521-1106 /FAX +55-19-3521-3023 (L.T.)
mhayashi@unifesp.br; Tel: +55-11-5576 4447/FAX +55-11-5576 4499 (M.A.F.H.)

Table S1. PLS-DA cross validation details obtained for the ^{1}H-NMR data on serum lipids of the animal model for SCZ (namely SHR) compared with control NWR strain.

**Measure**	**1 LV**	**2 LV**	**3 LV**	**4 LV**	**5 LV**	**6 LV**	**7 LV**
**Accuracy**	0.826	0.870	0.913	1.000	1.000	1.000	1.000
**R^2**	0.481	0.545	0.788	0.898	0.946	0.984	0.993
**Q^2**	0.229	0.323	0.628	0.808	0.860	0.956	0.962

LV - Latent Variable

Table S2. PLS-DA cross validation details obtained for the ^{1}H-NMR data on serum lipids from NWR after administration of pro-psychotic psychostimulants amphetamine (AMPH) or lisdexanfetamine (LDX), with doses of 0.5 and 5.0 mg/kg.

**Measure**	**1 LV**	**2 LV**	**3 LV**	**4 LV**	**5 LV**	***6 LV***	**7 LV**
**Accuracy**	0.148	0.230	0.344	0.541	0.525	0.656	0.738
**R^2**	0.128	0.680	0.758	0.804	0.878	0.899	0.934
**Q^2**	0.049	0.477	0.575	0.617	0.623	0.638	0.596

LV - Latent Variable

Table S3. PLS-DA cross validation details obtained for the ^{1}H-NMR data from SHR strain serum lipids after treatment with typical (haloperidol, HAL) or atypical (clozapine, CLZ) antipsychotic drugs.

Measure	1 LV	2 LV	3 LV	4 LV	5 LV	6 LV	7 LV	8 LV
**Accuracy**	0.667	0.697	0.909	0.939	0.909	0.970	1.000	1.000
**R2**	0.082	0.381	0.732	0.839	0.918	0.964	0.974	0.984
**Q2**	8.730	0.214	0.579	0.710	0.743	0.867	0.896	0.924

LV - Latent Variable

Table S4. PLS-DA cross validation details obtained for the ^{1}H-NMR data from control NWR strain serum lipids after treatment with typical (haloperidol, HAL) or atypical (clozapine, CLZ) antipsychotic drugs.

Measure	1 LV	2 LV	3 LV	4 LV	5 LV	6 LV	7 LV	8 LV
**Accuracy**	0.647	0.941	0.941	0.941	0.971	1.000	1.000	1.000
**R2**	0.395	0.508	0.600	0.896	0.946	0.980	0.988	0.998
**Q2**	0.084	0.390	0.496	0.711	0.868	0.955	0.973	0.988

LV - Latent Variable

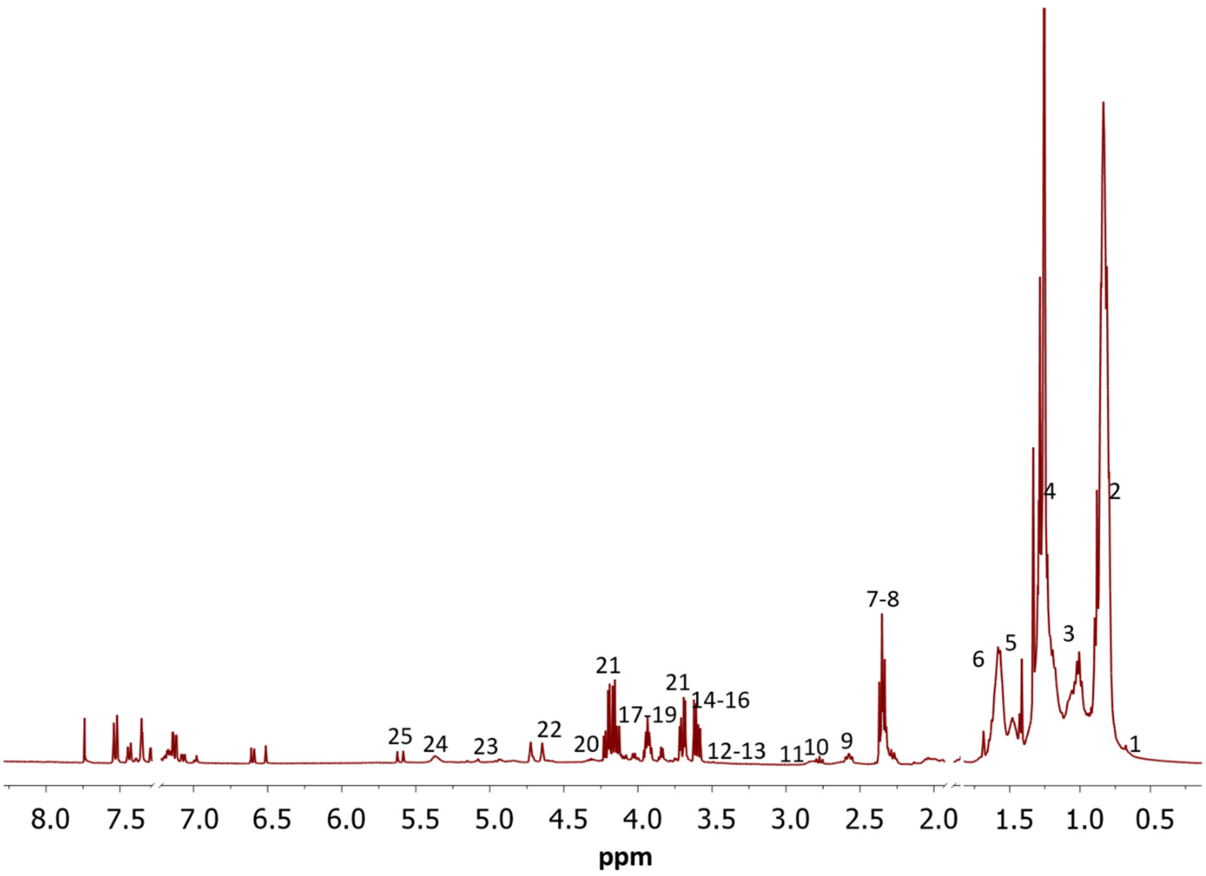

Figure S1. Representative ¹H-NMR spectrum of neutral lipids isolated from animal serum sample. Chemical shifts, peak multiplicity, and coupling constants for the 1-25 compounds were checked against databases and lipids NMR libraries. Assignment and numbers presented are detailed in the main text.

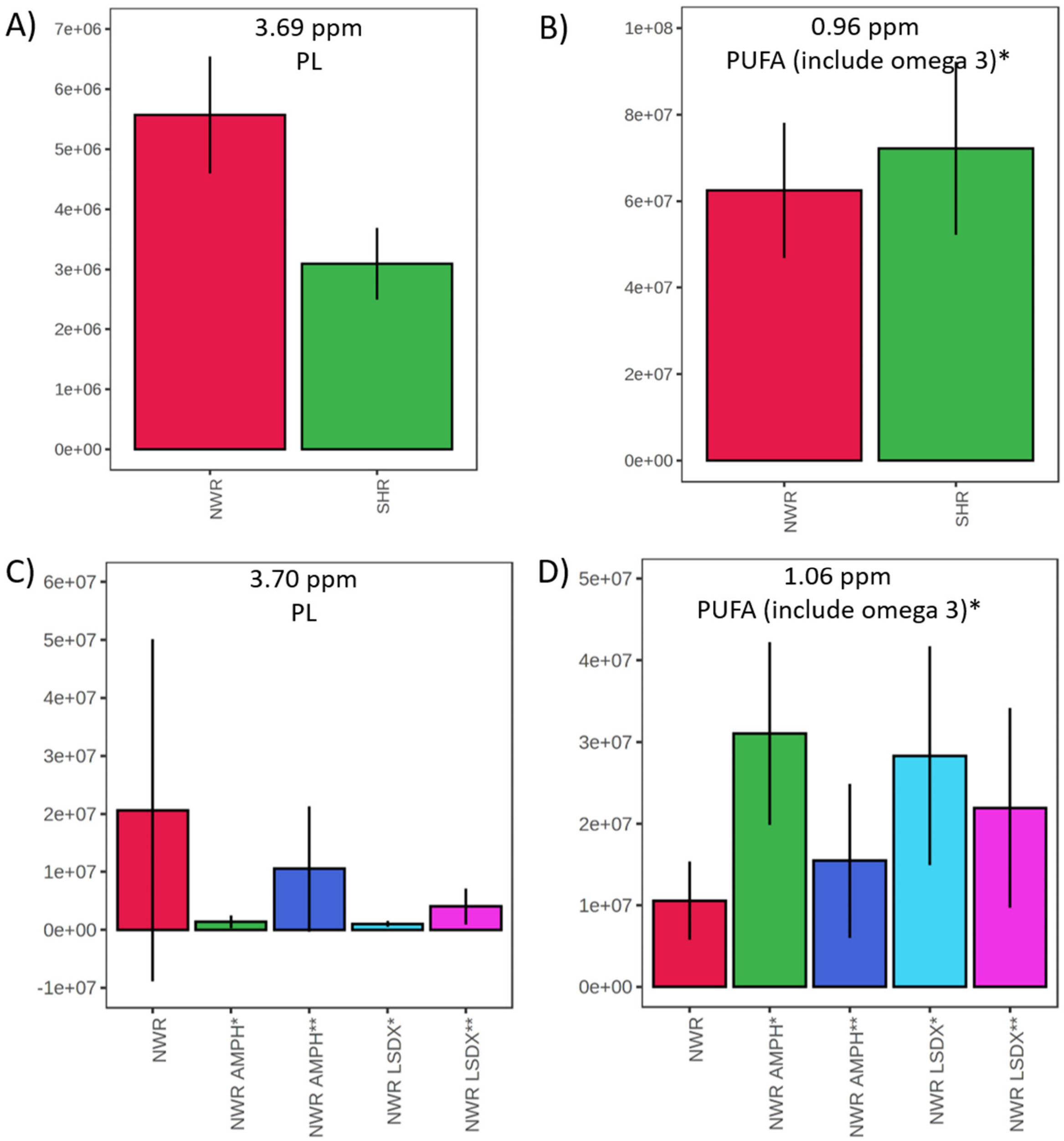

Figure S2. Graphical summary (mean ± SD) for the variation of some lipids in animal serum samples. A) polar lipids (PL) in SHR and NWR; B) apolar lipids, such as linolenic acid, omega-3 (Ln), in SHR and NWR, C) polar lipids, such as linoleic acid, omega-6 (L), in NWR after acute administration of psychostimulants LSDX or AMPH; D) apolar lipids, such as linolenic acid, omega-3 (Ln), in NWR after acute administration of psychostimulants LSDX or AMPH, using two different doses: *0.5 mg/kg and **5.0 mg/kg.

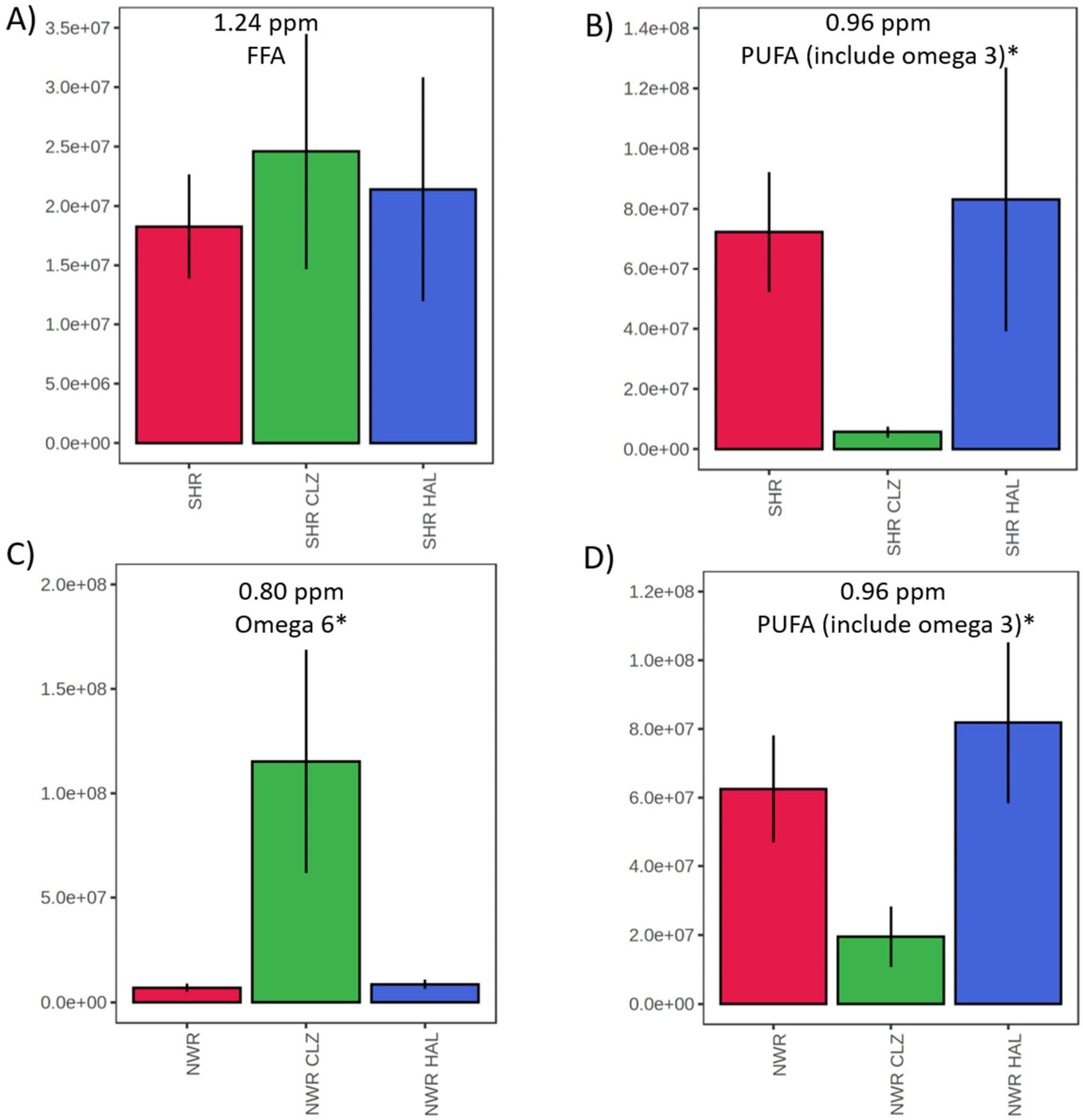

Figure S3. Graphical summary (mean ± SD) for variations of some lipids in animal serum samples after long-term treatment with antipsychotics - typical haloperidol (HAL) or atypical clozapine (CLZ): A) free fatty acids (FFA) in SHR, B) omega-3 in SHR, C) omega-6 in NWR, and D) omega-3 in NWR. * Other free fatty acids and polyunsaturated fatty acids (PUFA) are present.

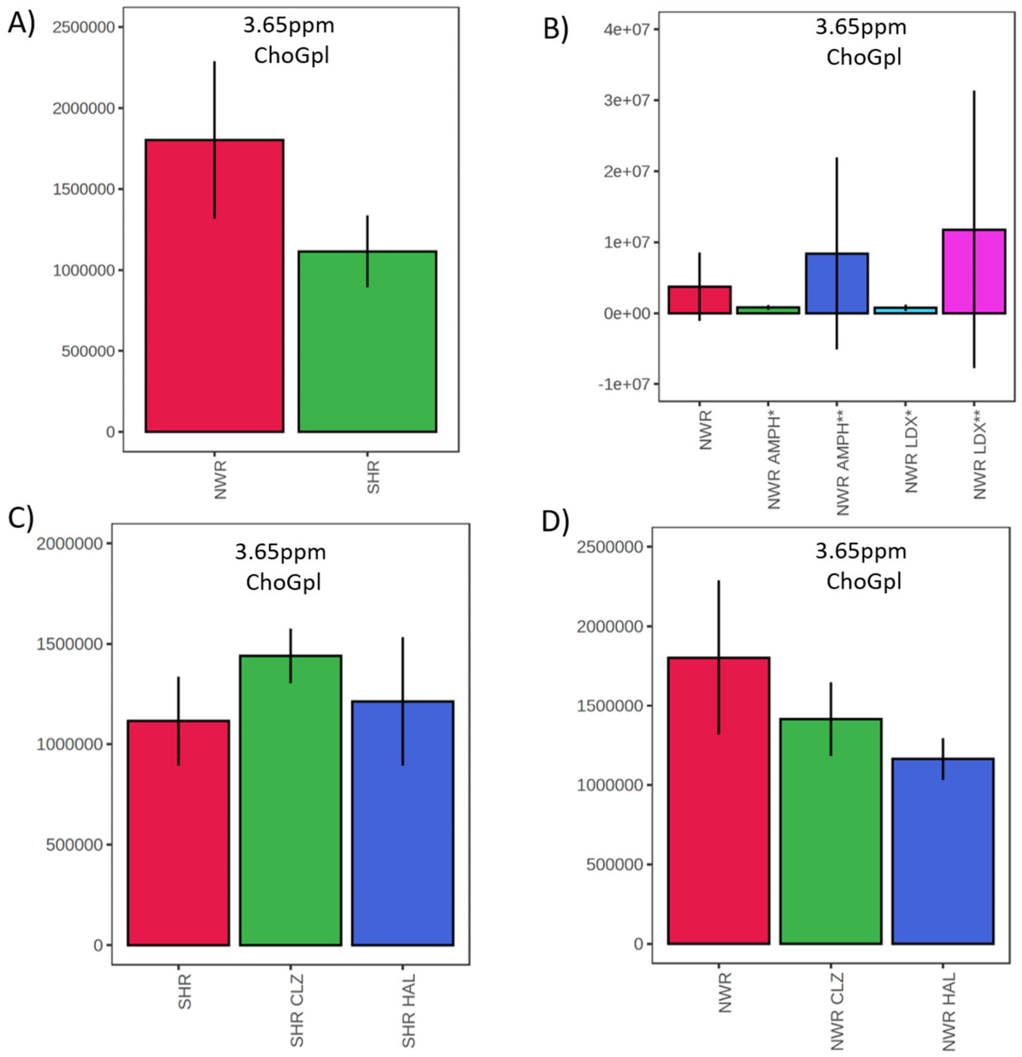

Figure S4. Graphical summary (mean ± SD) for choline glycerophospholipids (ChoGpl) in investigated animal serum samples. A) NWR and SHR, B) NWR after acute administration of psychostimulants LSDX or AMPH (*0.5 mg/kg and **5.0 mg/kg), C) SHR after treatment with antipsychotics - clozapine (CLZ) or haloperidol (HAL), D) NWR after long-term treatment with antipsychotics - typical haloperidol (HAL) or atypical clozapine (CLZ).
